# Seahorses of the *Hippocampus coronatus* complex: taxonomic revision, and description of *Hippocampus haema*, a new species from Korea and Japan (Teleostei, Syngnathidae)

**DOI:** 10.3897/zookeys.712.14955

**Published:** 2017-10-31

**Authors:** Sang-Yun Han, Jin-Koo Kim, Yoshiaki Kai, Hiroshi Senou

**Affiliations:** 1 Department of Marine Biology, Pukyong National University, Busan, South Korea; 2 Maizuru Fisheries Research Station, Field Science Education and Research Center, Kyoto University, Nagahama, Maizuru, Kyoto, Japan; 3 Kanagawa Prefectural Museum of Natural History, Kanagawa, Japan

**Keywords:** Genetic distance, morphology, molecular systematics, Pacific Ocean, taxonomy

## Abstract

Morphological and molecular analyses were conducted on 182 specimens belonging to the *Hippocampus coronatus* complex (*H. coronatus* sensu lato), collected in Korea and Japan 1933–2015, in order to clarify the taxonomic status of the species within this complex. Three species are recognized based on the shape of the coronet, the number of trunk rings (TrR) and tail rings (TaR), and presence or absence of a wing-tip spine (WS) at the dorsal fin base. *Hippocampus coronatus* Temminck & Schlegel, 1850 (*H. coronatus* sensu stricto), is diagnosed by 10 TrR, 37–40 TaR, an extremely high coronet (55.7–79.0 % head length) with four tips on the corona flat (CoT), and one WS. *Hippocampus sindonis* Jordan & Snyder, 1901 is diagnosed by 10 TrR, 35–38 TaR, a moderately high coronet (36.3–55.4 % HL) with five CoT, and no WS. A new species, *H. haema* is described on the basis of 140 specimens, characterized by 10 TrR, 35–38 TaR, a moderately high coronet (34.1–54.9 % head length) with four CoT, and two WS. *Hippocampus haema* is only known from the Korea Strait, western Kyushu, and East/Japan Sea. Recognition of the three species is supported by differences in mitochondrial DNA fragments (cytochrome *b*, 16S rRNA, and 12S rRNA).

## Introduction

The seahorse genus *Hippocampus* (Teleostei: Syngnathidae) exhibits a wide range of inter- and intra-specific variation, for example in skin filaments, color, and body proportions. Therefore, taxonomic relationships within *Hippocampus* have been controversial (Lourie et al. 1999, 2016), and more than 140 species have been named within this genus (Lourie et al. 2016; Eschmeyer et al. 2017). For example, Lourie et al. (2016) reviewed the genus and considered 41 species as valid, while Kuiter (2009) recognized *ca.* 79 valid species. Six species of *Hippocampus* have been recorded from Korea and Japan, viz., *H. coronatus* Temminck & Schlegel, 1850, *H. mohnikei* Bleeker, 1853, *H. histrix* Kaup, 1856, *H. kuda* Bleeker, 1852, *H. trimaculatus* Leach, 1814, and *H. sindonis* Jordan & Snyder, 1901. Another two species, *H. kelloggi* Jordan & Snyder, 1901 and *H. bargibanti* Whitley, 1970, were only recorded from Japan (Choi et al. 2002; Lourie et al. 2004; Kim et al. 2005; Senou et al. 2006; Kim et al. 2013; Senou 2013; Lourie et al. 2016).

The species (or species group) *H. coronatus* sensu lato has been defined by possessing ten trunk rings, 34–40 tail rings, a bony armor, double gill openings (Lourie et al. 1999, 2004; Kim et al. 2005; Kuiter 2009; Foster and Gomon 2010; Senou 2013; Lourie 2016), and a tall coronet on the head, which exhibits a wide range of height variation (Jordan and Snyder 1901; Mitani 1956; Lourie et al. 1999, 2004). Some authors have stated that this group includes two species, *H. coronatus* (sensu stricto), which has an extremely high coronet and a snout length ~2.33 times the head length, and *H. sindonis*, which has a moderately high coronet and a snout length ~3 times the head length (Jordan and Snyder 1901; Okada and Matsubara 1938; Matsubara 1955; Lourie et al. 1999; Senou 2002; Lourie et al. 2004; Senou 2013), while others considered the variation in coronet height only as intraspecific variation (Mitani 1956; Araga 1984; Senou 1993). Based on variation in mitochondrial DNA (partial 12S rRNA), Mukai et al. (2000) suggested that the *H. coronatus* complex (*H. coronatus* sensu lato) consists of two genetically diverged groups.

Although the Korean seahorse (Korean name: *Haema*) has been identified as *H. coronatus* (Mori 1928; Chyung 1977; Kim et al. 2001; Kim et al. 2005), the height of its coronet and the number of tail rings appear to agree better with that described for *H. sindonis* (Jordan and Snyder 1901; Lourie et al. 1999, 2004; Kim et al. 2013; Senou 2013; Han et al. 2014). In fact, *H. sindonis* has often been confused with *H. coronatus* (Lourie et al. 1999, 2016), and the height of the coronet in the type series of *H. coronatus* varies (Boeseman, 1947). These controversies have contributed to the uncertainty about the distribution of *H. coronatus* in both Korea and Japan, and led to its classification in the Data Deficient (DD) category of the International Union for Conservation of Nature and Natural Resources (IUCN) Red List, as there is a lack of information on population trends (Zhang and Pollom 2016). The present study aims to clarify the taxonomic status of Korean seahorses, redescribing *H. coronatus* and *H. sindonis* and describing a new species, all belonging to the *H. coronatus* complex.

## Materials and methods

### Material examined

**Figure 1.**
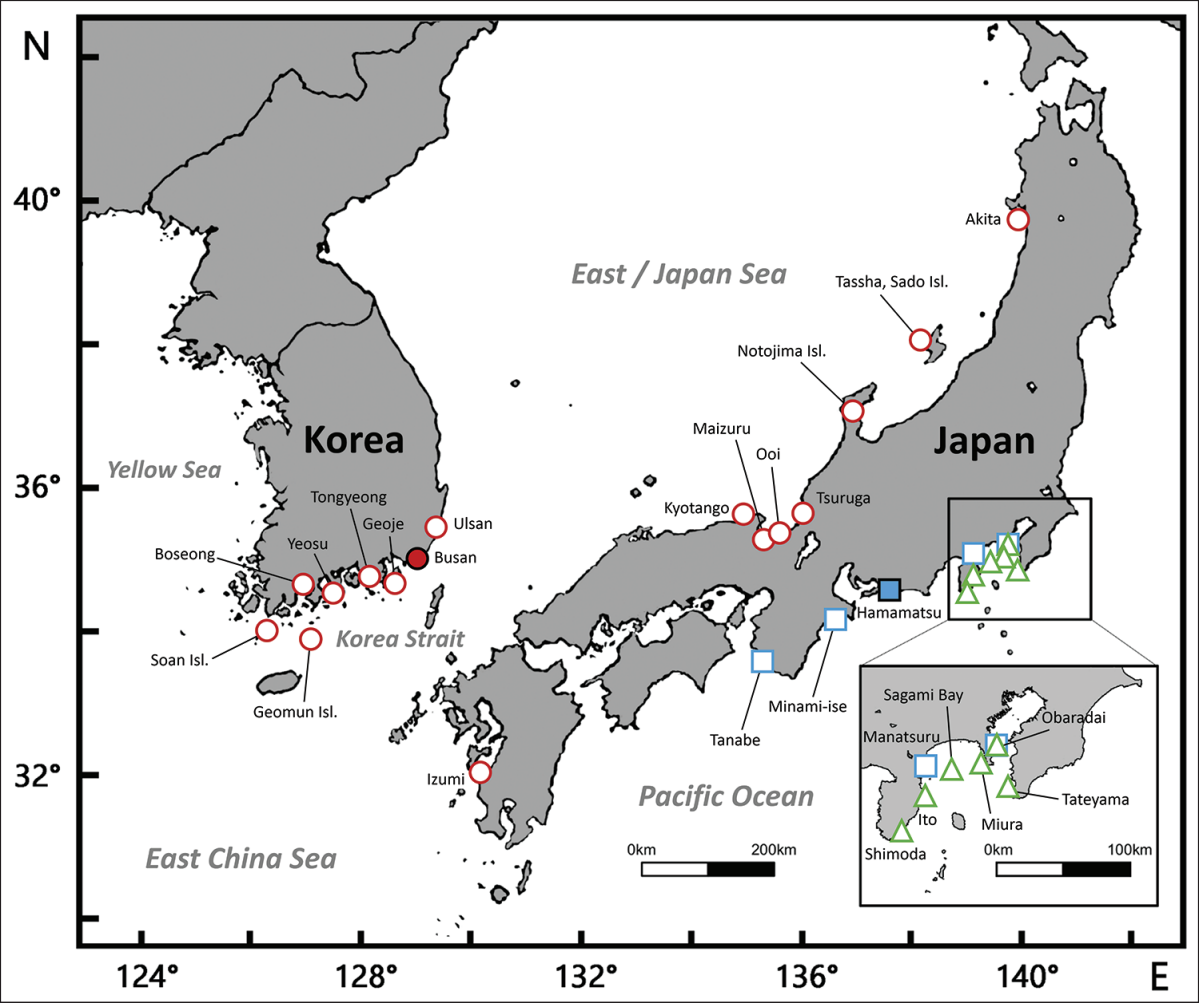
Distribution of the species within the *Hippocampus coronatus* complex: *H. haema* (red circles; the filled red circle indicates the holotype), *H. coronatus* (green triangles), and *H. sindonis* (blue squares; the filled blue square indicates the holotype).

A total of 182 specimens of *H. coronatus* sensu lato collected from Korean and Japanese waters (Fig. 1) were subjected to morphological analyses. Voucher specimens were deposited in Korea [Department of Marine Biology, Pukyong National University (**PKU**); National Institute of Biological Resources (**NIBR**)], Japan [Maizuru Fisheries Research Station, Field Science Education and Research Center, Kyoto University (**FAKU**); Kagoshima University Museum (**KAUM**); Kanagawa Prefectural Museum of Natural History (**KPM**)], Europe [Naturalis Biodiversity Center (**RMNH**), The Netherlands], and the United States [Smithsonian National Museum of Natural History (**USNM**)].

### Morphological analysis

**Figure 2.**
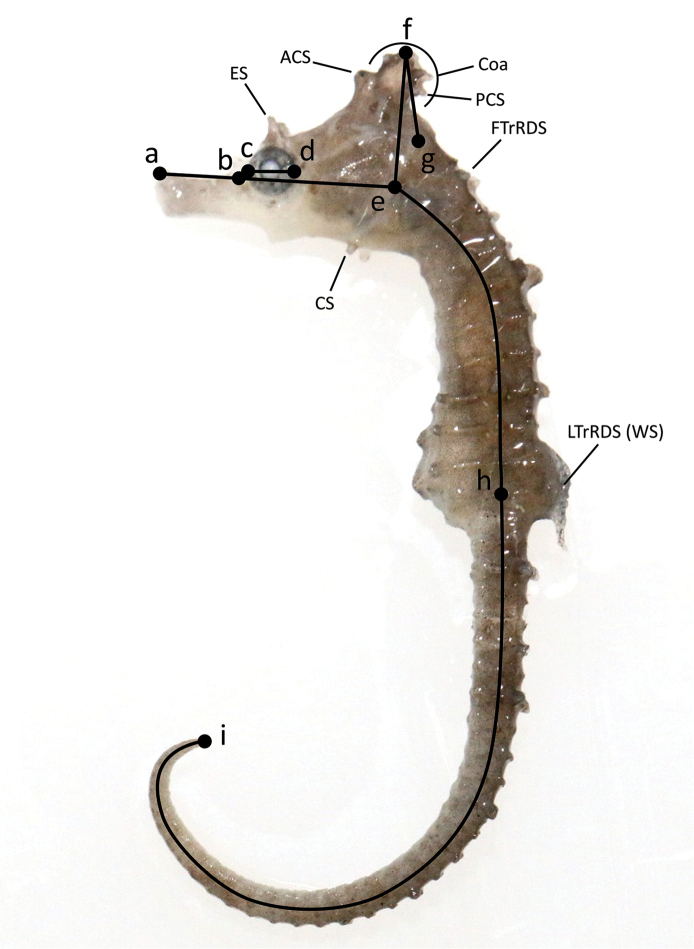
Meristic and morphometric characters used in *Hippocampus* analyses following Lourie (2003). Abbreviations: eye spine (ES), cheek spine (CS), anterior coronet spine (ACS), posterior coronet spine (PCS), corona (Coa), dorsal spine of the first trunk ring (FTrRDS), dorsal spine of the last trunk ring (LTrRDS; Wing-tip spine [WS] as in *H. coronatus* and *H. haema*). Points used for measurements: **a** tip of snout (upper jaw) **b** anterior side of tubercle/spine **c** anterior edge of orbit **d** posterior edge of orbit **e** mid-point of cleithral ring **f** median groove (central depression) of coronet **g** gill opening **h** mid-point of lateral ridge of the last trunk ring **i** tail tip. Measurements: **a–b** snout length (SnL) **c–d** eye diameter (ED), **a–e** head length (HL) **e–f** coronet height from mid-point of cleithral ring (CHMC) **f–g** coronet height from gill opening (CHGO) **e–h** trunk length (TrL) **h–i** tail length (TaL) **a–e–h–i** standard length (SL). Photographed specimen *H. haema* PKU 10129 (paratype).

Procedures used for counts and measurements follow Lourie (2003) and are presented in Fig. 2.

**Morphological terms are abbreviated as**:

**TrR** trunk rings

**TaR** tail rings

**DsR** TrR and TaR supporting the dorsal fin

**D** dorsal fin rays

**A** anal fin rays

**P** pectoral fin rays

**CS** cheek spine below the operculum

**ES** eye spine above the eye

**FTrDS** first TrR dorsal spine

**LTrDS** last TrR dorsal spine

**WS** wing-tip spine: a thick-recurved spine on dorsal fin base as in *H. coronatus* and *H. haema*

**ACS** anterior coronet spine

**PCS** posterior coronet spine: 5^th^ tip on corona flat

**Coa** corona: posterior crest of coronet

**CoT** number of tips on corona flat

**Measurements are abbreviated as**:

**SL** standard length

**HL** head length

**CHGO** coronet height from gill opening to the median groove on corona (along central depression between 1^st^ and 2^nd^ tip on it)

**CHMC** coronet height from mid-point of cleithral ring to the median groove on corona

**SnL** snout length

**ED** eye diameter

**TrL** trunk length

**TaL** tail length

Meristic data were obtained from soft X-rays of the 182 *H. coronatus* sensu lato specimens. Measurements were obtained using the microscope-integrated Active Measure software (Shinhanoptics, Seoul, Korea). The coronet height was measured as CHMC (Lourie 2003) and CHGO (Temminck and Schlegel 1850; Jordan and Snyder 1901) (Fig. 2) so that our results could be compared to those reported in previous studies (Temminck and Schlegel 1850; Jordan and Snyder 1901; Lourie et al. 1999). Sexual dimorphism analysis was conducted on the 152 adults (80 females and 72 males). These are all the specimens over 53.9 mm, which is the minimum SL at maturation defined for *H. coronatus* sensu lato (Choi et al. 2006).

### Molecular analysis

Tissue from the right eye ball or from the right-side of the tail was used to isolate genomic DNA from 22 specimens with moderately high coronets, collected in Busan, Tongyeong, Boseong, Soan Island, Maizuru, and Minami-ise, and from four specimens with extremely high coronets collected in Miura. Isolation was performed using an AccuPrep® Genomic DNA Extraction Kit (Bioneer, Daejeon, Korea), according to the manufacturer’s instructions.

Three partial mitochondrial DNA loci (cytochrome *b* [cyt *b*], 16S rRNA, and 12S rRNA) were amplified via polymerase chain reaction (PCR), which was conducted on an S1000™ Thermal Cycler (Bio-Rad, Hercules, CA, USA). The PCR solutions consisted of 3 μl 10× Ex Taq buffer (20 mM Mg^2+^ plus), 2.4 μl 2.5 mM dNTPs, 1 μl each primer, 0.1 μl TaKaRa Ex Taq DNA polymerase (Takara Bio, Kusatsu, Shiga, Japan), 3 μl genomic DNA, and distilled water to bring the total volume to 30 μl. The PCR amplification of cyt *b* was conducted using primers Shf2 (5'-TTGCAACCGCATTTTCTTCAG-3') and Shr2 (5'-CGGAAGGTGAGTCCTCGTTG-3') under the following conditions: initial denaturation at 94°C for 2:30 min; 35 cycles of denaturation at 94°C for 30 s, annealing at 50°C for 30 s, and extension at 72°C for 1:15 min; final extension at 72°C for 5 min (Lourie and Vincent 2004). Using the universal primers 16Sal-L (5'-CGCCTGTTTATCAAAAACAT-3') and 16Sbr-H (5'-CCGGTCTGAACTCAGATCACGT-3'), 16S rRNA was amplified as follows: initial denaturation at 94°C for 5 min; 35 cycles of denaturation at 94°C for 30 s, annealing at 50°C for 1 min, and extension at 72°C for 1 min; final extension at 72°C for 10 min (Palumbi 1996). The amplification of 12S rRNA was conducted using primers OMT16SF (5'-TGCCAGCCACCGCGGTTATACCT-3') and tRNA02 (5'-GGATGTCTTCTCGGTGTAAG-3') (both from Mukai et al. 2000), under the following conditions, which were modified from Mukai et al. (2000): initial denaturation at 95°C for 2:30 min; 30 cycles of denaturation at 95°C for 1 min, annealing at 55°C for 1 min, and extension at 70°C for 2 min; final extension at 70°C for 5 min. Amplified PCR samples were purified using a Davinch™ PCR Purification Kit (Davinch-K, Seoul, Korea), according to the manufacturer’s instructions. Sequencing reactions were performed in a DNA Engine Tetrad 2 Peltier Thermal Cycler (Bio-Rad) using an ABI BigDye(R) Terminator 3.1 Cycle Sequencing Kit (Applied Biosystems, Waltham, MA, USA).

Sequences of the three gene regions belonging to members of the *H. coronatus* complex (*H. coronatus* and *H. sindonis*), its sister species (*H. mohnikei*), some members of the *H. kuda* complex (*H. kuda*, *H. reidi*, and *H. ingens*) (Lourie et al. 1999, 2004), and one outgroup (*Syngnathus schlegeli*) were retrieved from the GenBank database (www.ncbi.nlm.nih.gov) (Table 1). Sequences obtained for each species were concatenated and each gene region was treated as a partition. To compare our results with that of Mukai et al. (2000), an additional analysis focusing on 12 rRNA sequence variation was performed. GenBank sequences were aligned with those obtained in the present study using BioEdit7 (Hall 1999), and pairwise genetic distances were calculated using the Kimura 2-parameter model (Kimura 1980) on MEGA6 (Tamura et al. 2013). Neighbor-joining (NJ) trees were constructed in MEGA6, and confidence levels were assessed using 1000 bootstrap replications.

**Table 1.** GenBank accession numbers and sources of the mitochondrial gene sequences used in the evaluation of the phylogenetic relationships among species belonging to the *Hippocampus coronatus* complex.

Species	Locus	Accession No.	Source
*Hippocampus haema* sp. n.	cyt *b*	KP744863–>KP744882	Present study
	16S rRNA	KP744883–>KP744902	
	12S rRNA	KP744903–>KP744922	
*H. coronatus*	cyt *b*	KT167545–>KT167548	Present study
	16S rRNA	KT167549–>KT167552	
	12S rRNA	KT167553–>KT167556	
	12S rRNA	AB032030	Mukai et al. (2000)
*H. sindonis*	cyt *b*	KT167539–>KT167540	Present study
	16S rRNA	KT167541–>KT167542	
	12S rRNA	KT167543–>KT167544	
	12S rRNA	AB032029	Mukai et al. (2000)
*H. mohnikei*	complete mitogenome	KT780446	Zhang et al. (2017)
	12S rRNA	AB032028	Mukai et al. (2000)
*H. kuda*	complete mitogenome	AP005985	Kawahara et al. (2008)
*H. reidi*	complete mitogenome	KJ123692	Wang et al. (2016)
*H. ingens*	complete mitogenome	KF680453	Zhang et al. (2015)
*Syngnathus schlegeli*	complete mitogenome	AP012318	Song et al. (2014)

## Systematics

### Hippocampus coronatus

Taxon classificationAnimaliaSyngnathiformesSyngnathidae

Temminck & Schlegel, 1850

**Figure 3.**
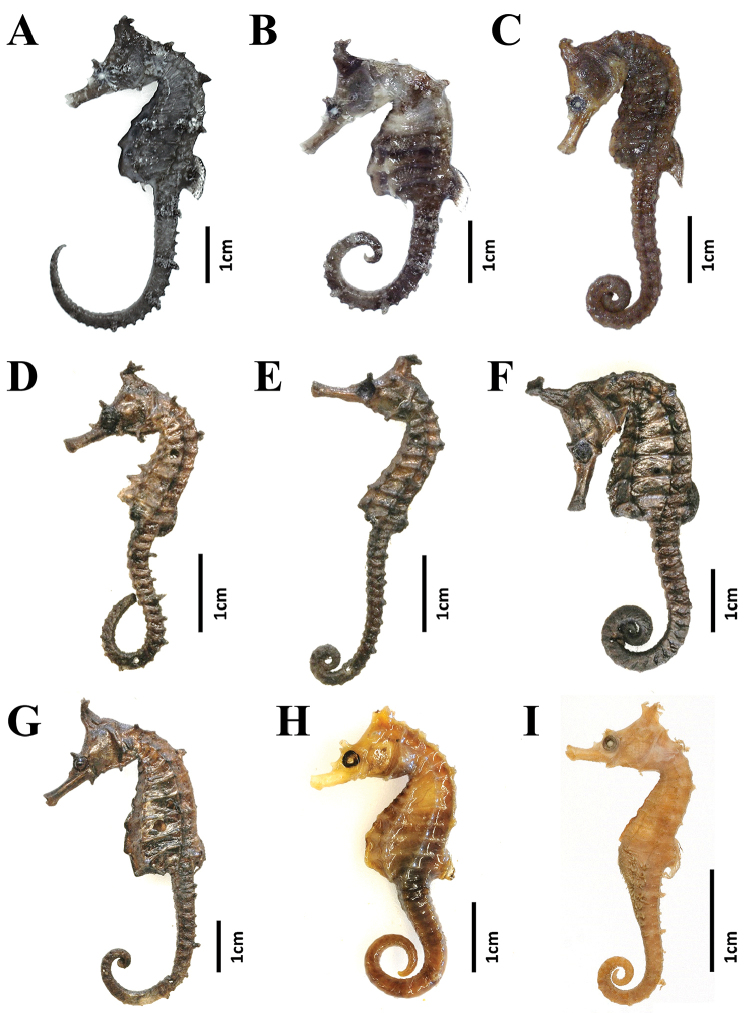
Specimens within the *Hippocampus coronatus* complex examined in the present study. **A–E** *H. haema* **A** PKU 9641 (holotype, Busan, Korea) **B** FAKU 135644 (paratype, Maizuru, Japan) **C** KPM-NI 24769 (paratype, Akita, Japan) **D** RMNH.PISC.D 1541 (paratype, Japan) **E** RMNH.PISC.D 1542 (paratype, Japan) **F–G** *H. coronatus* **F** RMNH.PISC.D 1543 (lectotype, Japan) **G** RMNH.PISC.D 1544 (paralectotype, Japan) **H–I** *H. sindonis* **H** RMNH.PISC 3924 (Japan) **I** USNM 49730 (holotype, Hamamatsu, Japan).

**Figure 4.**
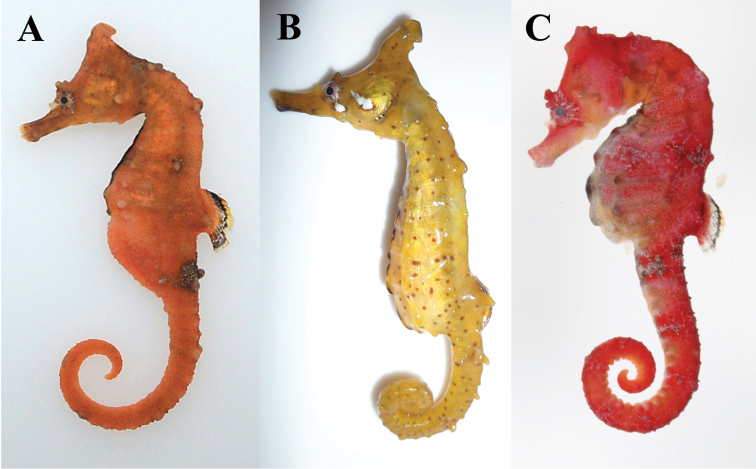
Coloration of fresh specimens. **A** *Hippocampus haema* (paratype, PKU 9424) **B** *H. coronatus* (FAKU 137351) **C** *H. sindonis* (FAKU 137339).

**Figure 5.**
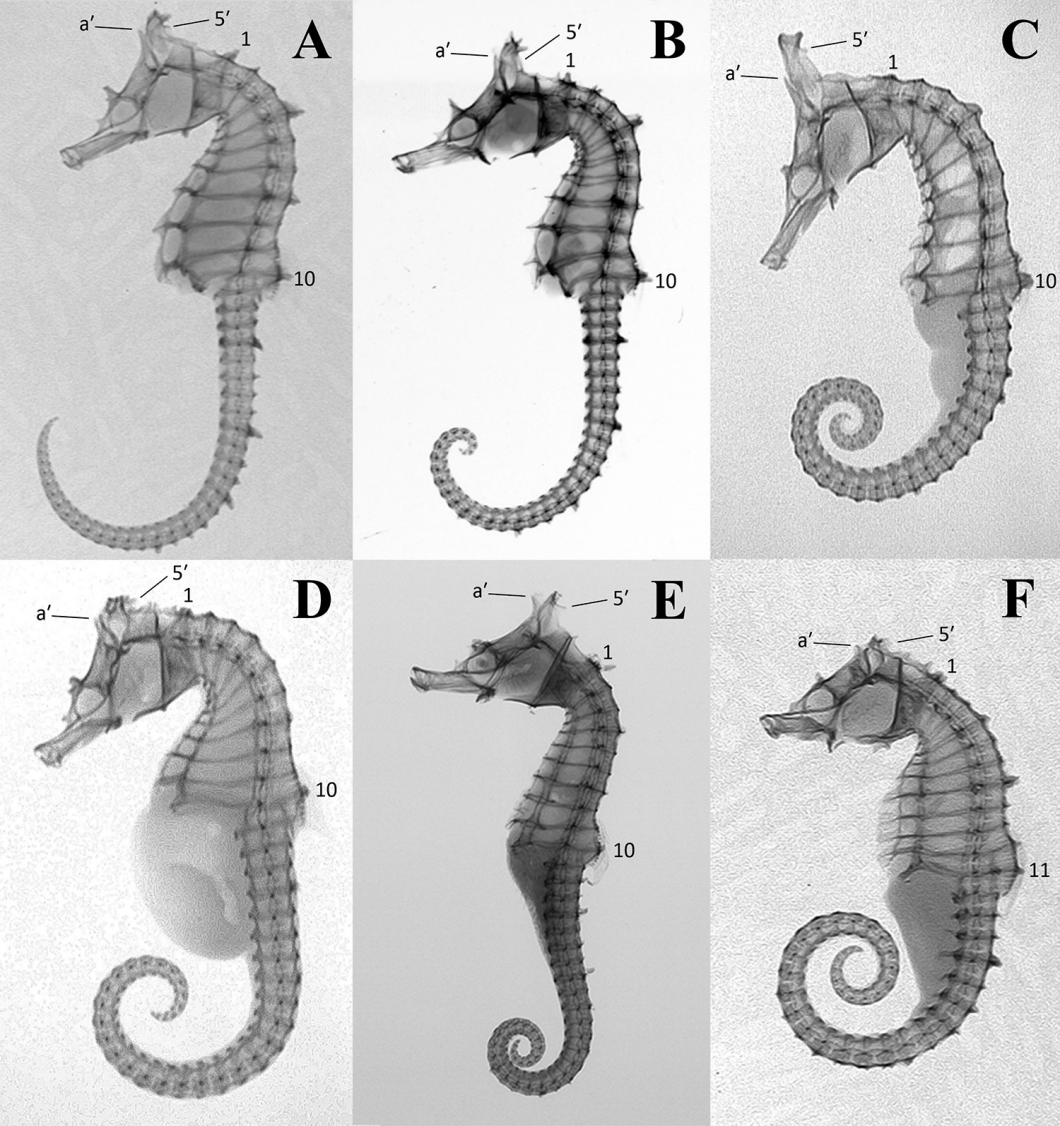
X-radiographs of *Hippocampus* specimens. **A** *H. haema* PKU 9641 (holotype) **B** *H. haema* NIBR-P 5412 (paratype) **C** *H. coronatus* FAKU 137348 **D** *H. sindonis* FAKU 137340 **E** *H. sindonis* USNM 49730 (holotype) **F** *H. mohnikei* FAKU 135643. a' indicates the anterior coronet spine; 5' indicates the posterior coronet spine (the 5^th^ tip on the corona); the first (1) and last (10 or 11) trunk rings are marked.

**Figure 6.**
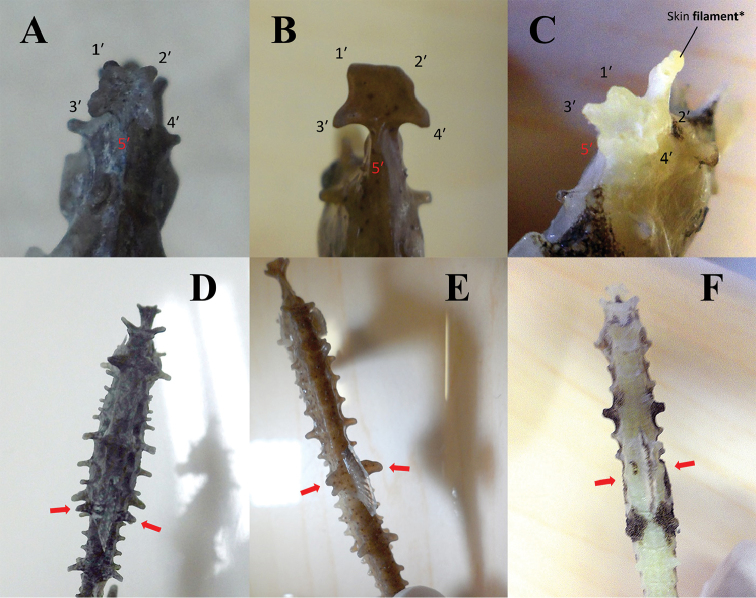
Distinctive morphological characters among species within the *Hippocampus coronatus* complex. **A–C** Tips on the corona flat **A** *H. haema* (PKU 9641, holotype) **B** *H. coronatus* (KPM-NI 7720) **C** *H. sindonis* (KPM-NI 19797). Numbers indicate coronet tips; the 5^th^ coronet tip (posterior coronet spine) is indicated in red. The * indicates the appendage growing on the anterior coronet spine, which is a skin filament **D–F** Dorsal fin base spines (red arrows; wing-tip spines in **D** and **E**) **D** *H. haema* (PKU 9641, holotype) **E** *H. coronatus* (KPM-NI 7720) **F** *H. sindonis* (KPM-NI 19797).

Figs 3F–G , 4B , 5C , 6B , 6E , Tables 2 , 3

Hippocampus coronatus Temminck and Schlegel 1850: 274, pl. 120 (fig. VII) (Lectotype: RMNH.PISC.D 1543; Paralectotype: RMNH.PISC.D 1544; type locality: Japan; Boeseman 1947: 196); Kaup 1853: 229; Jordan and Snyder 1901: 18; Matsubara 1955: 431; Jordan et al. 1913: 100; Boeseman 1947: 195; Burgess and Axelrod 1972: 212; Araga 1984: 89; Senou 1993: 489, 1294; Lourie et al. 1999: 88; Mukai et al. 2000: 139; Senou 2000: 536; Senou 2002: 536, 1508; Lourie et al. 2004: 42; Yoshino and Senou 2008: 76; Kuiter 2009: 129; Kohno et al. 2011: 127; Senou 2013: 635, 1911; Lourie 2016: 106; Lourie et al. 2016: 21.

#### Material examined.

**Japan**. RMNH.PISC.D 1543 (lectotype of *H. coronatus*, photograph from RMNH), female, 103.3 mm SL, von Siebold collection. RMNH.PISC.D 1544 (paralectotype of *H. coronatus*, photograph from RMNH), female, 100.2 mm SL, von Siebold collection. FAKU 137348–137351, 4, 96.4–112.6 mm SL, Miura, Kanagawa, Nov 2014, H. Sugawara. KAUM-I 20721, 1, 73.7 mm SL, Takane, Hamasa, Tateyama, Chiba, 34°58'38"N; 139°47'19"E, depth 20 m, 2 Dec 2008, M. Aizawa. KPM-NI 1375, 1, 82.0 mm SL, 6 Sep 1964. KPM-NI 7301–7302, 2, 110.5–117.9 mm SL, depth 4 m, 12 Jul 2000; KPM-NI 7535, 1, 124.1 mm SL, 19 Dec 2000, S. Gosho; KPM-NI 7718–7720, 3, 113.1–115.6 mm SL, depth 1–12 m, 18 Jan 2001, S. Gosho; KPM-NI 8075, 1, 115.3 mm SL, depth 3–4 m, 26 Jul 2001, K. Uchino & D. Kanbayashi; in front of Misaki Marine Biological Station, The University of Tokyo, Aburatsubo Bay, Koajiro, Miura, Kanagawa. KPM-NI 14854, 1, 24.1 mm SL, in front of Keikyu Aburatsubo Marine Park, Koajiro, Miura, Kanagawa, T. Mukai. KPM-NI 19270, 1, 113.7 mm SL, Cape of Manazuru, Obaradai, Yokosuka, Kanagawa, 1 Jul 2000, T. Yokoo. KPM-NI 19272, 1, 108.5 mm SL, Kannonzaki, Tatara-hama, Obaradai, Yokosuka, Kanagawa, 12 Dec 1998, T. Yokoo. KPM-NI 18765, 18772, 2, 27.8–28.4 mm SL, 14 Jun 2006, Y. Miyazaki; KPM-NI 21540, 1, 39 mm SL, 6 Jul 2003; KPM-NI 21541, 1, 53.4 mm SL; 19 Jun 2004; KPM-NI 25371, 1, 103.5 mm SL, depth 7 m, 27 Jun 2009, S. Shimizu; in front of Tateyama Station of Field Science Center, Tokyo University of Marine Science and Technology, Banda, Tateyama, Chiba. KPM-NI 27901–27903, 3, 51.4–67.5 mm SL, 2–6m depth, 5 Oct 2010, N. Takeuchi; KPM-NI 29380, 1, 47.8 mm SL, depth 2–6 m, 3 Jun 2011, N. Takeuchi; Gouchome, Shimoda, Shizuoka. KPM-NI 30596, 1, 133.0 mm SL, Sagami bay, Kanagawa Hadano High School, Kanagawa.

#### Diagnosis.

A species of *Hippocampus* having a bony body; double gill openings; ring (R: TrR + TaR) 10 + 37–40, mode 10 + 39 (lectotype: 10 + 38); extremely high coronet, straight or inclined backwards; CoT 4; CHGO 43.0–60.1 % HL; CHMC 55.7–79.0 % HL; WS thick and recurved.

#### Description.

Head and trunk folded at approximately right angle; snout elongated and fused; pelvic and caudal fins absent; prehensile tail; D 12–15, mode 14 (lectotype: 14); A 4; P 10–13, mode 12 (lectotype: 12); D always greater than or equal to P; CS 1; ES 1; SnL 35.6–44.2 % HL; ED 32.3–62.9 % SnL; HL 56.6–71.3 % TrL; TrL 42.6–64.5 % TaL; flat and smooth skin generally covering armor-plated body; ACS degenerative; Coa expanded; CoT 4 arising from degenerative PCS; WS two fused LTrRDS (lower more developed than upper and recurved; upper LTrRDS occasionally standing out [Fig. 6E]); dorsal and lateral spines more prominent on 1^st^, 4^th^, 7^th^, and 10^th^ TrR than on other TrRs, except occasionally for lateral spines on 10^th^ TrR, occasionally; usually no skin filaments on body, but, occasionally, a strand was observed on ACS or on the forward part of Coa; blunt (or absent) body spine; often whitish radial blotches from iris to surrounding eye and striped-pattern body; occasionally semicircular band present on dorsal fin; variable color, light to dark red-brown or yellow, sometimes showing numerous thin whitish striations and/or dark small dots along body; male brood pouch sometimes speckled with fine white and dark spots (Kuiter 2009); no particular sexual dimorphism, apart from male brood pouch.

#### Distribution.

Southeastern coast of Honshu (Japan), from Izu Peninsula (Shizuoka Prefecture) to Boso Peninsula (Chiba Prefecture) (Fig. 1). *Hippocampus coronatus* lives in weed habitats, especially in floating *Sargassum* (Kuiter 2009; Senou 2013), within shallow areas (0–20 m depth).

#### Etymology.

The Latin word *coronatus* means crowned. The new Korean name, *Wanggwan-haema* means ‘crowned seahorse’, in agreement with the English and scientific names. In fact, *Haema*, which has the connotation ‘common’ and ‘fish species belonging to the genus *Hippocampus*’ in Korean, has been used to name seahorses commonly found in Korea, whereas *Wanggwan-haema* has been informally used to refer to *H. coronatus* in Korean. In addition, the word *wanggwan* [crown] is more suited for *H. coronatus*, whose coronet is considerably higher than that of *H. haema*. The Japanese name *Tatsu-no-otoshigo* literally means ‘dragon’s bastard child’.

#### Remarks.

**Figure 7.**
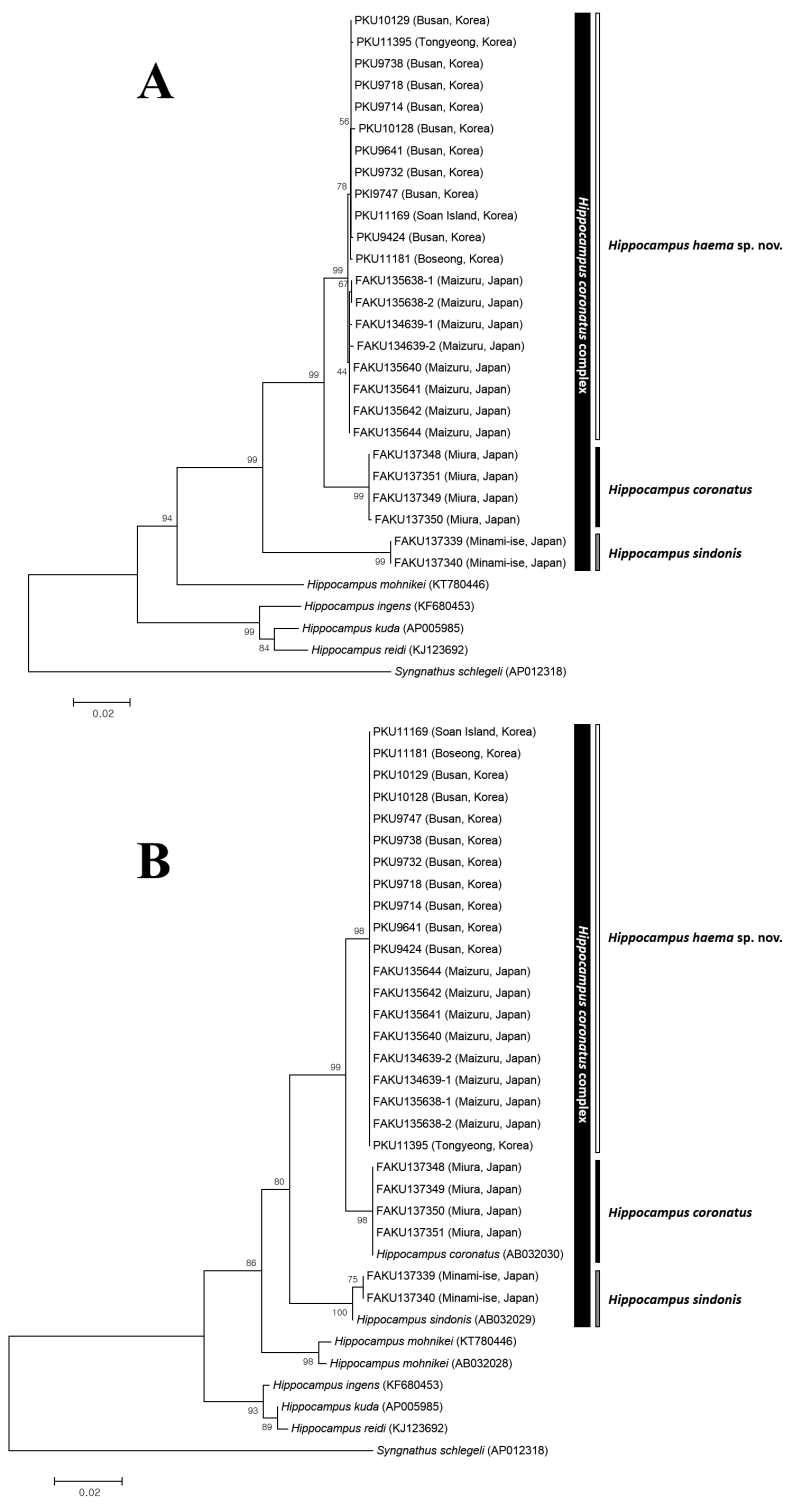
Neighbor-joining tree showing the relationships among species of *Hippocampus* based on mtDNA sequences. **A** tree produced using multiple loci (cytochrome *b*, 16S rRNA, and 12S rRNA) as partitions **B** tree produced using 12S rRNA, only. Numbers in branches indicate bootstrap probabilities obtained from 1000 bootstrap replications. Scale bar = genetic distance of 0.02.

Temminck and Schlegel (1850) described *H. coronatus* based on five specimens. Boeseman (1947) designated one of these specimens RMNH.PISC.D 1543 as the lectotype. As a consequence the other three specimens RMNH.PISC.D 1541, RMNH.PISC.D 1542, and RMNH.PISC.D 1544 became paralectotypes, except that RMNH.PISC 3924 was reidentified as *H. mohnikei* (see remarks of *H. sindonis* below). However, two of the specimens described in Boeseman (1947), RMNH.PISC.D 1541 and 1542, have a moderately high coronet, not agreeing with the *H. coronatus* described in the present study and being more similar to *H. haema* (see species description below). The lectotype RMNH.PISC.D 1543 and the paralectotype RMNH.PISC.D 1544 have an extremely high coronet, which agrees with the present description of *H. coronatus*. Our 28 specimens have an extremely high coronet, a wing-tip spine on the dorsal fin base, and CoT 4, as described and illustrated in Temminck and Schlegel (1850). The phylogenetic trees obtained in the present study also support the differentiation of these 28 specimens from *H. sindonis* and *H. haema* (Fig. 7).

The type series does not match Temminck and Schlegel (1850)’s description on the basis of five dried specimens and an illustration which was based on a small male seahorse (Temminck and Schlegel 1850; Kaup 1853). The lectotype (RMNH.PISC.D 1543) and the paralectotype (RMNH.PISC.D 1544) are large female seahorses (100.2–103.3 mm SL), and RMNH.PISC.D 1541, 1542, and RMNH.PISC 3924 are small female seahorses (67.5–74.0 mm SL). RMNH.PISC 3924 is preserved in spirits unlike the other specimens, therefore Boeseman’s inclusion of this sample is questionable. The original illustration of *H. coronatus* from Temminck and Schlegel (1850) might be the missing fifth dry specimen (personal communication, M. van Oijen).

The type locality of *H. coronatus* has not been established. Although it is thought to be Nagasaki (Eschmeyer et al. 2017), no specific locality information is provided for the type series or in previous studies (Temminck and Schlegel 1850; Boeseman 1947; Lourie et al. 1999). Seahorses are used historically as charm for safe-birth in East Asia (Korea, Japan, and China) and as a trinket in western culture (Lourie et al. 1999; Scales 2009). Thus, we cannot exclude the possibility that dried specimens might be from someone’s folkloric collection (MacLean, 1973). This historical element might support that the type series was not caught in the Nagasaki area. Therefore, it is possible that collectors not only gathered specimens from Nagasaki, but Edo (present-day Tokyo) as well, which is the habitat of *H. coronatus* in this study (see Fig. 1; personal communication, M. van Oijen; MacLean 1973; Compton and Thujsse 2013; Nofuji et al. 2013).

Although *H. coronatus* sensu stricto was considered to be distributed along the coast of Japan and southern coast of Korea, we only found records from the Pacific Ocean. Mori (1928) reported *H. coronatus* off Korea for the first time, but the original data consisted only of checklists, not providing descriptions; thus, Mori (1928) might be reporting the occurrence of *H. haema* or *H. coronatus*. Therefore, the distribution of *H. coronatus* needs to be reviewed. In Korea and Japan, seahorse identification has been generally treated as a laborious task, leading to taxonomic controversy and misidentifications; thus, we recommend a careful revision of *H. coronatus* recorded from Korea and Japan.

Senou (2002) and (2013) suggested that the publication date for *H. coronatus* was in 1847. However, based on Sherborn and Jentick (1895), Boeseman (1947), Mees (1962), Bauchot et al. (1982), and Eschmeyer et al. (2017), the year should be 1850.

### Hippocampus sindonis

Taxon classificationAnimaliaSyngnathiformesSyngnathidae

Jordan & Snyder, 1901

Figs 3H–I , 4C , 5D–E , 6C , 6F , Tables 2 , 3

Hippocampus sindonis Jordan and Snyder 1901: 17, pl. 11 (Holotype: USNM 49730; type locality: Totomi bay, off Hamamatsu, Totomi Province, Shizuoka, Japan); Jordan et al. 1913: 100; Matsubara 1955: 431; Araga 1984: 89; Lourie et al. 1999: 119; Mukai et al. 2000: 139; Senou 2000: 536; Senou 2002: 536, 1508; Lourie et al. 2004: 74; Yoshino and Senou 2008: 76; Kuiter 2009: 131; Senou 2013: 635, 1911; Lourie 2016: 108; Lourie et al. 2016: 39.
Hippocampus coronatus : Burgess and Axelrod 1972: 211; Araga 1984: 89; Senou 1993: 489 (left fig.), 1294 (non Temminck & Schlegel).
Hippocampus mohnikei : Jordan and Snyder 1901: 18; Jordan et al. 1913: 98; Boeseman 1947: 196; Matsubara 1955: 431; Burgess and Axelrod 1972: 210; Araga 1984: 89 (non Bleeker).
Hippocampus japonicus : Burgess and Axelrod 1972: 211 (non Kaup).

#### Material examined.

**Japan**. USNM 49730 (holotype of *H. sindonis*, photograph and radiograph from USNM), male, 49.1 mm SL, Totomi bay, off Hamamatsu, Totomi Province, Shizuoka, dredged by the U.S. Fish Commission Steamer *Albatross* (Jordan and Snyder 1901). RMNH.PISC 3924 (photograph from RMNH), 1 female, 74.0 mm SL. FAKU 121388, 1, 69.4 mm, Tanabe, Wakayama, Jan 1969. FAKU 137339, 1 93.0 mm, Hozaura, Minami-ise, Watarai, Mie, depth 20–25 m, Nov 2014, H. Sugawara. FAKU 137340, 1, 95.9 mm, Nayaura, Minami-ise, Watarai, Mie, depth 25–30 m, Mar 2014, H. Sugawara. KPM-NI 19257, 1, 59.4 mm SL, 16 May 1999, D. Sugita; KPM-NI 19258, 1, 44.4 mm SL, 18 Oct 1997, M. Kojima; KPM-NI 19259, 1, 30.1 mm SL, 5 Jul 1998, T. Kamano; KPM-NI 19261, 1, 43.3 mm SL, 7 Aug 1998, N. Ogata; KPM-NI 19262, 1, 32.2 mm SL, 25 Aug 1998, N. Ogata; Kannonzaki, Tatara-hama, Obaradai, Yokosuka, Kanagawa. KPM-NI 19475, 1, 82.1 mm SL, 23 Sep 2007 K. Okubo; KPM-NI 19797–19798, 2, 75.1–99.8 mm SL, 18 Oct 2007, K. Okubo; KPM-NI 21947, 1, 75.4 mm SL, K. Okubo; Manatsuru, Ashigarashimo, Kanagawa.

#### Diagnosis.

A species of *Hippocampus* having a bony body; double gill openings; R 10 + 35–38 (holotype: 10 + 37); coronet moderately high; CoT 5; CHGO 26.8–41.0 % HL; CHMC 36.3–55.4 % HL; a very blunt or truncated spine on the dorsal fin base; no WS on dorsal fin base.

#### Description.

Head and trunk folded at approximately right angle; snout elongated and fused; pelvic and caudal fins absent; prehensile tail; D 11–15, mode 12 (holotype: 15); A 4; P 11–14, mode 11 (holotype: 14); D always greater than or equal to P; CS 1; ES 2 (anterior ES smaller than posterior ES); SnL 28.7–37.2 % HL; ED 41.5–69.0 % SnL; HL 57.2–80.1 % TrL; TrL 38.3–52.1 % TaL; coarse skin often covering armor-plated body; moderately high coronet; CoT, 5; body spines blunt, truncated, or absent; spines on 1^st^, 4^th^, 7^th^, and 10^th^ TrR more prominent than on other TrRs, except for the lateral spine on the 10^th^ TrR; several skin filaments on ACS and ES, and prominent TrR and TaR spines, or skin filaments absent on these structures; variable coloration on fresh specimens, including white, red, yellow, brown, and grey; variable patterns on fresh specimens, often presenting white radial blotches on iris and surrounding eye, stripes and/or blotches on body, and, occasionally, a semicircular stripe on dorsal fin; preserved specimens, black, pale white, brown, or grey; no sexual dimorphism apart from male brood pouch.

#### Distribution.

Southeastern coast of Honshu (Japan), from Tanabe (Wakayama Prefecture) to Boso Peninsula (Chiba Prefecture) (Fig. 1). *Hippocampus sindonis* lives in a wide range of habitats, from shallow high-energy algae reefs to soft bottom habitats (Kuiter 2009), at 2–30 m depth (Senou 2013).

#### Etymology.

The specific name *sindonis* was derived from the name of M. Sindo, an assistant curator of fishes at Stanford University (Jordan and Snyder 1901; Lourie 2016). The English name was coined by Kuiter (2009). The Japanese name *Hanatatsu* literally means ‘*hana* (flower or blossom, which indicates gorgeous) + *tatsu* (dragon, or the abbreviation of the word “*Tatsu-no-otoshigo*: seahorse”)’, and refers to the beautiful color and skin filaments of the species.

#### Remarks.

The 14 Japanese specimens of *H. sindonis* have a moderately high coronet with five CoT, and a couple of prominently blunted or truncated spines on the dorsal fin base, therefore corresponding to the description and holotype of *H. sindonis* provided by Jordan and Snyder (1901). In the 12S rRNA tree, our *H. coronatus* specimens (voucher number: FAKU 137348–137351) appeared in the same clade as Mukai et al.'s (2000) high coronet specimen (GenBank accession number AB032030) whereas our *H. sindonis* specimens (voucher numbers FAKU 137339–137340) formed a clade with Mukai et al.'s (2000) low coronet specimen (accession number AB032029) (Fig. 7B). *Hippocampus sindonis* is considered the most external group within the *H. coronatus* complex because of its homogenous CoT (= 5) and no WS, as found in *H. coronatus* complex outgroups (e.g., *H. mohnikei* and *H. trimaculatus*).

RMNH.PISC 3924 was labeled ‘Hippocampus fasciatus Kaup 1853’ (Boeseman 1947), which is a nomen nudum in *Hippocampus*. Boeseman (1947) noted that RMNH.PISC 3924 was related to *H. coronatus* and *H. mohnikei*, and that its morphology agreed with Jordan and Snyder’s (1901) description as well as with Bleeker’s (1853) *H. mohnikei* specimens. However, we found that Bleeker’s *H. mohnikei* (RMNH.PISC 7259, 3 specimens) differ from RMNH.PISC 3924 in their TrR number (11 in Bleeker’s specimens vs. 10 in RMNH.PISC 3924). Thus, RMNH.PISC 3924 belongs to the *H. coronatus* complex, and its ES 2 and coronet features (moderately high coronet with 5 CoT) allow identifying it as *H. sindonis*. Jordan and Snyder (1901) stated that *H. sindonis* was distinguished from *H. mohnikei* by dorsal fin features (D 15 and long dorsal fin base in *H. sindonis* vs. D 11–13 and short dorsal fin base in *H. mohnikei*), but their key did not consider individual variations. Our *H. sindonis* specimens agree with both *H. mohnikei* and *H. sindonis* descriptions, but the paradoxical inconsistency between the original description and type series of *H. mohnikei* requires a further taxonomic review of this species, and, therefore, we compared our specimens with ‘*H. mohnikei*’ holotype and not to the original description of the species (Lourie et al. 1999; Eschmeyer et al. 2017).

Nakamura (1999a) described a single specimen of *H. sindonis* caught off Kumamoto, Japan, which is questionable, as there are no other records of *H. sindonis* from western Kyushu. This record may have been based on *H. haema* because spines were not mentioned in Nakamura’s description. Kim et al. (2013) recorded a *H. sindonis* specimen from Korean waters (voucher: NIBR-P 5412; Fig. 5B). However, the morphology of this specimen indicates that it rather belongs to *H. haema* and we include it in the type series of *H. haema*. Thus, there are no reliable records of *H. sindonis* from Korea.

### Hippocampus haema sp. n.

Taxon classificationAnimaliaSyngnathiformesSyngnathidae

http://zoobank.org/13F12FB3-B435-4AD4-B02F-110E20C06C56

Figs 3A–E , 4A , 5A–B , 6A , 6D , Tables 2 , 3

Hippocampus coronatus : Jordan and Snyder 1901: 19; Mori 1928: 5; Boeseman 1947: 195; Mitani 1956: 30; Chyung 1977: 272; Araga 1984: 89; Senou 1993: 489 (right fig.), 1294; Kim and Lee 1995: 76; Nakamura 1999b: 125; Senou 2000: 536; Choi et al. 2002: 141; Senou 2002: 536, 1508; Kim et al. 2005: 203; Choi et al. 2006; Yoshino and Senou 2008: 76; Kohno et al. 2011: 127; Senou 2013: 635, 1911; Han et al. 2014: 423 (non Temminck & Schlegel).
Hippocampus cf. coronatus : Kuiter 2009: 128.
Hippocampus sindonis : Nakamura 1999a: 124; Yoshino and Senou 2008: 76; Kim et al. 2013: 42 (non Jordan & Snyder).
Hippocampus kuda : Kim et al. 2001: 67, Myoung et al. 2002: 74 (non Bleeker).
Hippocampus sp.: Kim and Ryu 2017: 110.

#### Holotype.

PKU 9641, 1, female, 90.3 mm SL, Namcheon Harbor, Namcheon 1-dong, Suyeong-gu, Busan, Korea, 35°08'16"N; 129°06'51"E, 9 Aug 2013, H. J. Kwun, hand net.

#### Paratypes.

139 specimens: specimens (74.0–99.0 mm SL). **Korea**: NIBR-P 5412, 1, female, 74.0 mm SL, off Geomun Island, Yeosu-si, Jellanam-do, depth 18 m, 17 Apr 2009, T. S. Park, SCUBA Diving & hand net. NIBR-P 1602, 1, 59.4 mm SL, Wonpo, Yeosu-si, Jeollanam-do, 27 Aug 2006, J. H. Ryu. NIBR-P 19724, 3, 58.4–71.8 mm SL, 25 Jan 2012, H. G. Cho & S. H. Lee; NIBR-P 19725–19727, 19729, 7, 33.3–102.2 mm SL, 13 Sep 2012, Y. Eun, S. Lee & S. S. Hong; Jisepo-ri, Irun-myeon, Geoje-si, Gyeongsangnam-do. PKU 6097, 1, 77.5 mm SL, 30 Aug 2011; PKU 9422–9424, 3, 80.6–92.3 mm SL, 12 Jul 2013, hand net; PKU 9704, 1, 82.3 mm SL, 1 May 2013, J. M. Lee; PKU 9705–9712, 8, 65.7–98.1 mm SL, 26 Jul 2012, J. M. Lee; PKU 9713–9717, 9719–9720, 7, 61.6–85.1 mm SL, 9 Dec 2012, J. M. Lee; PKU 9721–9723, 3, 80.8–91.5 mm SL, 20 Aug 2012, J. M. Lee; PKU 9724–9731, 8, 73.2–113.9 mm SL, 17 Jul 2012, J. M. Lee; PKU 9732–9740, 9, 62.4–100.2 mm SL, 17 Aug 2012, J. M. Lee; PKU 9741–9747, 7, 56.4–81.9 mm SL, 21 Jun 2012, J. M. Lee; PKU 9748, 1, 56.8 mm SL, 11 Sep 2012, J. M. Lee; PKU 10128–10129, 2, females, 52.2–62.7 mm SL, 23 Oct 2013, H. J. Kwun; PKU 54069–54074, 6, 32.3–77.5 mm SL, 21 Mar 2015, J. M. Lee; Namcheon Harbor, Namcheon 1-dong, Suyeong-gu, Busan, 35°08'16"N; 129°06'51"E, hand net. PKU 7230–7233, 4, 41.9–83.7 mm SL, Ulsan, 14 Sep 2012, hand net. PKU 10277, 1, 72.7 mm SL, Minrak Harbor, Millak-dong, Suyeong-gu, Busan, 35°09'14"N; 129°07'51"E, 20 Feb 2014, H. J. Yu & W. J. Lee, hand net. PKU 11159, 1, 30.9 mm SL, Hak-ri, Ilgwang-myeon, Gijang-gun, Busan, 22 Jul 2014, J. Y. Bae, hand net. PKU 11170–11180, 11, 74.2–102.4 mm SL, Soan Island, Soan-myeon, Wando-gun, Jeollanam-do, May 2014, S. Rho, bottom trawl. PKU 11181–11182, 2, 71.8–84.2 mm SL, Gunhak village, Jeonil-ri, Hoecheon-myeon, Boseong-gun, Jeollanam-do, 24 Dec 2013, S. Rho, bottom trawl. PKU 11266, 1, 74.1 mm SL, 24 Jul 2014; PKU 11634, 1, 69.9 mm SL, 25 Sep 2014; Hwayang-myeon, Yeosu-si, Jeollanam-do, hand net. PKU 11395–11401, 7, 62.3–98.7 mm SL, Jangu Island, Suwol-ri, Dosan-myeon, Tongyeong-si, Gyeongsangnam-do, Sep 2014, K. S. Han & H. D. Mun, Shrimp beam trawl. PKU 11449, 1, 81.0 mm SL, Jul 2014; PKU 11635–11637, 3, 15.9–84.7 mm SL, 24 Sep 2014; Gijang-gun, Busan, hand net. **Japan**: RMNH.PISC.D 1541–1542 (photograph by RMNH), 2, female, 67.5–68.5? mm SL, von Siebold collection. FAKU 109359, 1, 58.0 mm SL, Tassha, Sado Island, Niigata, 24 Oct 1955. FAKU 135638, 2, 82.7–88.9 mm SL, 22 Sep 2011; FAKU 135639, 2, 53.2–86.0 mm SL, 23 Aug 2010; FAKU 135640, 135644, 2, 76.2–86.8 mm SL, 29 Jul 2011; FAKU 135641, 1, 61.4 mm SL, 20 Aug 2008; FAKU 135642, 1, 57.4 mm SL, 6 Sep 2008; Maizuru Bay, Maizuru, Kyoto, Y. Kai. FAKU 136087, 1, 76.1 mm SL, Tsuruga, Fukui, 28 Jun 2014. FAKU 136119, 1, 59.2 mm SL, Kamai, Kyotango, Kyoto, 19 Jul 2014, F. Tashiro. KPM-NI 1615, 1, 91.6 mm SL, Aug 1933. KPM-NI 6770, 1, 57.9 mm SL, Azo, Tsuruga, Fukui, depth 5 m, 13 Aug 1999, T. Nomura. KPM-NI 24769, 1, female, 83.3 mm SL, Akita, H. Sugiyama. KPM-NI 31204, 1, 47.5 mm SL, Takahama-cho, Ooi, Fukui, 2 Oct 2012, M. Mune. KPM-NI 31620, 1, 72.7 mm SL, 27 Feb 2013; KPM-NI 31707, 1, 60.6 mm SL, 11 Mar 2013; Ogurui, Takahama-cho, Ooi, Fukui, depth 7 m, M. Mune. KPM-NI 31880–31883, 4, 76.6–85.5 mm SL, depth 0–2 m, 7 May 2013; KPM-NI 36111–36112, 2, 77.1–78.6 mm SL, depth 1–3 m, 28 Apr 2014; Agurizaki Point, Ooshima, Ooi-cho, Ooi-gun, Fukui, M. Mune. KPM-NI 35122–35123, 2, 46.3–46.8 mm SL, Tanoura, Takahama, Ooi-cho, Ooi, Fukui, depth 0–1 m, 3 Jul 2013, M. Mune. KPM-NI 35291–35297, 7, 54.5–74.9 mm SL, Koda Fishing Port, Notojimakouda-machi, Notojima Island, Nanao, Ishikawa, depth 1–3 m, 2013, H. Masaki. KAUM-I 12745, 1, 100.5 mm SL, 12 Oct 2007, depth 5 m, kept in Kagoshima Aquarium and dead on 8 Dec 2008; KAUM-I 12746, 1, 96.9 mm SL, 13 Feb 2008, kept in Kagoshima Aquarium and dead on 4 Aug 2008; off Nagashima Station, Faculty of Fisheries, Kagoshima University, Usui, Azuma, Izumi, Kagoshima, M. Yamada. KAUM-I 19885, 1, male, 99.0 mm SL, off Nagashima Station, Faculty of Fisheries, Kagoshima University, Usui, Azuma, Izumi, Kagoshima, 32°13'22"N; 130°10'31"E, 13 Feb 2008, Kagoshima Aquarium, hand net, kept in Kagoshima Aquarium and dead on 30 Apr 2007.

#### Diagnosis.

A species of *Hippocampus* having a bony body; double gill openings; R 10 + 35–38, mode 10 + 36 (holotype: 10 + 36); coronet moderately high and turned back on top; CoT 4; CHGO 22.7–41.6 % HL; CHMC 34.1–54.9 % HL; a WS on the dorsal fin base.

#### Description.

Head and trunk folded at approximately right angle; snout elongated and fused; pelvic and caudal fins absent; prehensile tail; D 11–14, mode 13 (holotype: 14); A 4; P 10–13, mode 12 (holotype: 13); D always greater than or equal to P; CS 1; ES 1–2 (in ES 2, anterior ES smaller than posterior ES), mode 1 (holotype: 2); SnL 28.8–49.0 % HL; ED 27.1–68.9 % SnL; HL 57.3–88.7 % TrL; TrL 37.4–57.2 % TaL; often flat and smooth skin covering armor-plated body; coronet turned back on top; CoT 4 arising from degenerative PCS (5^th^ coronet tip); WS two fused spines (lower spine more developed than upper spine, recurved; occasionally, upper spine stands out giving appearance of two dorsal fin base spines); dorsal and lateral spines at 1^st^, 4^th^, 7^th^, and 10^th^ TrR more prominent than on other TrRs, except for lateral spines on 10^th^ TrR (occasionally none or degenerative spine); Several skin filaments on body, ACS, and prominent dorsal and lateral spines on 1^st^, 4^th^, and 7^th^ TrR; Several colors when fresh: black, white, orange, yellow, magenta, claret, brown, grey with black, red, or white stripe, and frostlike whitish or grey striations along prominent TrR and TaR; whitish radial blotches from iris to surrounding eye often present; semicircular band on dorsal fin occasionally present; when fixed in alcohol, specimens become black, white, brown, and grey; blunt (or absent) body spine; no particular sexual dimorphism except for male brood pouch. Minimum size at sexual maturity, 53.9 mm SL in males.

#### Distribution.

Korea: southern and southeastern coasts of the Korean Peninsula (from Soan Island to Ulsan); Japan: western coast of Kyushu (western Kagoshima Prefecture), northwestern coast of Honshu (from Kyoto Prefecture to Akita Prefecture) (Fig. 1). Lives in floating *Sargassum* and weeds on shallow soft bottom habitats from 0–18 m depth (e.g. Kim et al. 2016).

#### Etymology.

The Korean word *Haema* means ‘seahorse’, which connotes ‘representative’ and ‘common’. Thus, the scientific and Korean names *Haema* were chosen to indicate that this seahorse is the one most commonly found in Korea. The Japanese name *Himetatsu* means ‘princess seahorse’ or ‘dwarf seahorse’, and refers to its lower coronet and smaller body compared to *H. coronatus*.

#### Remarks.

Temminck and Schlegel (1850) described the extremely high coronet as follows: coronet height (CHGO, based on the inquiry of type specimens and on Jordan and Snyder [1901]’s description) of *H. coronatus* is identical to its SnL, 1/5 shorter than remaining HL (i.e., 4/9 of HL). All *H. haema* specimens present a moderately high coronet (CHGO 22.7–41.6 % HL and CHMC 34.1–54.9 % HL) when compared to *H. coronatus* (extremely high coronet, CHGO 43.0–60.1 % HL and CHMC 55.7–79.0 % HL). Our *H. sindonis* specimens (including the holotype, USNM 49730) differ from *H. haema* in their 5 CoT and blunt or truncated LTrDS (vs. CoT 4 and WS [recurved LTrDS] in *H. haema*) (Fig. 6). The genetic distance between *H. haema* and *H. coronatus* is greater than that between species of the *H. kuda* complex (i.e., *H. kuda*, *H. reidi*, and *H. ingens*), supporting specific distinctness (Fig. 7; Table 4).

Our data also suggest the existence of two subgroups, one from Korea and another from Japan: cyt *b* sequences of *H. haema* collected in these two areas consistently present two base pairs (bp) differences (0.3%–0.8% genetic distance). Based on molecular results, *H. haema* is more closely related to *H. coronatus* than to *H. sindonis* (Fig. 7; Table 4), but based on coronet height and on the number of TaR, except for CoT and WS, it is more similar to *H. sindonis* (Tables 2 and 3).

*Hippocampus haema* was collected off the southern and southeastern coasts of Korea, but we were not able to collect *H. haema* off the western or northeastern coasts of Korea; only *H. mohnikei* was collected from all Korean waters. A few studies have reported *H. coronatus* from the western coast of Korea (Lee and Seok 1984; Hwang 1998; Hwang et al. 1998; Hwang et al. 2005), but these publications are mostly checklists, similar to that of Mori (1928), and *H. mohnikei* is not referred to in written records. Such inconsistency might be the result of misidentifications. The northern boundaries of *H. coronatus* in Korean waters determined in our study are similar to the distributions found by Choi et al. (2002) and Kim et al. (2005), who stated *H. coronatus* was limited to the southern coast of Korea, similarly to *H. mohnikei*. We found that the habitat of *H. haema* is affected by the Tsushima Warm Current (Briggs 1995; Nakabo 2009; Ishizu et al. 2017) and, therefore, *H. haema* might only rarely be found off the western and northeastern coasts of Korea.

## Discussion

The NJ trees based on cyt *b* (670 bp), 16S rRNA (405 bp), and 12S rRNA (344 bp) recovered three monophyletic groups within the *H. coronatus* complex, all supported by high bootstrap probabilities (Fig. 7): viz. *Hippocampus coronatus* group, *H. sindonis* group, and *H. haema* group. This evidence strongly supports the existence of three species, *H. coronatus*, H. cf. coronatus, and *H. sindonis*, as suggested by Kuiter (2009).

Lourie et al. (1999, 2004), based on the rings supporting the dorsal fin base (DsR), stated *H. coronatus* had ‘2 + 0 (TrR + TaR)’ and *H. sindonis* had ‘2 + 1’. However, Jordan and Snyder (1901) described *H. sindonis* as ‘2 + 0’ and *H. coronatus* as ‘2 + 1’, which is the reverse. Moreover, all species within the *H. coronatus* complex described in the present study include ‘2 + 0’ and ‘2 + 1’ forms (Table 2). Thus, DsR is an inappropriate characteristic to diagnose the species studied here. *Hippocampus coronatus* has only one supraorbital spine whereas *H. sindonis* has two and *H. haema* has either one or two spines. Many ichthyologists have attempted to distinguish *H. coronatus* and *H. sindonis* based on color and skin filaments (especially Jordan and Snyder 1901). However, Curtis (2006) refuted the use of skin filaments on its key to distinguish *H. hippocampus* from *H. guttulatus*, as skin filaments grow irregularly in both species. Lourie et al. (1999) and Szabó et al. (2011) also suggested that color and skin filaments were affected by environment and/or growth, and therefore should be considered of limited diagnostic value. In the present study, several color and skin filament patterns were found in *H. haema*, which is in agreement with Mitani’s (1956) data for specimens sampled from Maizuru Bay, Japan. This author interpreted these as intraspecific variations, but, given the results obtained in this study by molecular analyses, we do not agree that *H. coronatus* and *H. sindonis* should be treated as a single species.

**Table 2.** Meristic and morphometric characters assessed in the species comprising the *Hippocampus coronatus* complex.

	*H. haema* sp. n.	*H. coronatus*			*H. sindonis*			
	Present study	Present study	Temminck and Schlegel (1850)	Jordan and Snyder (1901)	Lourie et al. (1999)	Present study	Jordan and Snyder (1901)	Lourie et al. (1999)
N	140	28	5	–	7	14	1	6
SL (mm)	15.9–113.9	24.1–133.0	?–127.0	90.0–115.0	–	30.9–108.3	38.0	–
**Counts**								
TrR	10	10	–	10	10	10	10	10
TaR	35–38 (36)	37–40 (39)	–	38–40	38–40 (39)	35–38 (36)	37	36–38 (37)
DsR	2 + 0, 2 + 1	2 + 0, 2 + 1	–	2 + 1	2 + 0	2 + 0, 2 + 1	2 + 0	2 + 1
D	11–14 (13)	12–15 (14)	–	13–14	14	11–15 (12)	15	11–15 (12)
A	4	4	–	–	–	4	–	–
P	10–13 (12)	10–13 (12)	–	11	12	11–14 (11)	14	12–14
CS	1	1	–	–	1	1	–	1
ES	1–2 (1)	1	–	–	1	2	2	2
WS	1	1	1	–	1	0	–	0
CoT	4	4	4	–	–	5	–	–
**Measurements**								
% HL								
CHGO	22.7–41.6 (32.2)	43.0–60.1 (51.6)	44.4	42.9	–	26.8–41.0 (33.9)	35.7	–
CHMC	34.1–54.9 (44.5)	55.7–79.0 (67.4)	–	–	–	36.3–55.4 (45.9)	–	–
SnL	28.8–49.0 (38.9)	35.6–44.2 (39.9)	44.4	42.9	40.0–43.4 (41.7)	28.7–37.2 (33.0)	35.7	30.3–35.7 (33.0)
% SnL								
ED	27.1–68.9 (48.0)	32.3–62.9 (47.6)	–	33.3	–	41.5–69.0 (55.3)	57.1	–
% TrL								
HL	57.3–88.7 (73.0)	56.6–71.3 (64.0)	–	60.0–66.7 (63.4)	–	57.2–80.1 (68.7)	75.0	–
% TaL								
TrL	37.4–57.2 (47.3)	42.6–64.5 (53.6)	–	50.0–71.4 (60.7)	–	38.3–52.1 (45.2)	50.0	–

N (number of samples), SL (standard length), TrR (trunk rings), TaR (tail rings), DsR (rings supporting dorsal fin), D (dorsal fin rays), A (anal fin rays), P (pectoral fin rays), CS (cheek spine), ES (eye spine), WS (wing-tip spine on dorsal fin base), CoT (tips on corona flat), HL (head length), CHGO (coronet height from gill opening), CHMC (coronet height from mid-point of cleithral ring), SnL (snout length), ED (eye diameter), TrL (trunk length), TaL (tail length). Bracket represents mode in counts and median in measurements

*Hippocampus coronatus* is ranked as DD in the IUCN Red List due to the lack of information on its population trends and to the uncertainty of its distributions, originating from taxonomic controversies (Zhang and Pollom 2016). *Hippocampus sindonis* is ranked as Least Concern (LC) because no major threat has been reported for its distribution (Fritzsche et al. 2010). The distribution of *H. coronatus* is similar to that of *H. sindonis* (i.e., southeastern coast of Honshu, Japan), and there is no data supporting its potential threat with distribution uncertainty. However, *H. coronatus* distribution has a narrower range than that of *H. sindonis* (Fig. 1), so it is more likely to be affected by human pressure. For these reasons, *H. coronatus* will likely be ranked above or equal to *H. sindonis* after further surveys of its population trends. To improve the conservation of these species, a better taxonomic understanding is required to resolve the DD rank of *H. coronatus* regarding the uncertainty of its distribution, as well as more data on its biology, habitat, and abundance. Previous studies considering the biology of *H. coronatus* conducted on local Korean areas (Choi et al. 2006, 2012; Huh et al. 2014; Park and Kwak 2015), might, in fact, indicate the biology of *H. haema*. Overfishing could potentially threat *H. haema* due to by-catch, given the species low density and patchy distribution (Choi et al. 2012; Zhang and Pollom 2016), and its wide distribution requires the study of populations across the entire area.

**Table 3.** Frequency distribution of meristic counts among species within the *Hippocampus coronatus* complex. Holotypes and lectotypes are marked by an asterisk.

	**Tail rings**						
	**35**	**36**	**37**	**38**	**39**	**40**	***N***
*Hippocampus haema* sp. n.	17	53*	50	18			138
*H. coronatus*			1	9*	15	3	28
*H. sindonis*	4	4	4*	2			14
	**Dorsal fin rays**						
	**11**	**12**	**13**	**14**	**15**		***N***
*H. haema*	1	22	89	28*			140
*H. coronatus*		1	6	18*	3		28
*H. sindonis*	1	8	1	3	1*		14
	**Pectoral fin rays**						
	**10**	**11**	**12**	**13**	**14**		***N***
*H. haema*	6	45	65	24*			140
*H. coronatus*	2	4	18*	4			28
*H. sindonis*		7	5	1	1*		14

**Table 4.** Pairwise genetic distances between *Hippocampus* species and the outgroup *Syngnathus schlegeli* based on multiple loci (cytochrome *b*, 16S rRNA, and 12S rRNA) and on 12S rRNA only. Asterisks indicate intraspecific pairwise distances calculated from one base pair difference.

Multiple loci	1	2	3	4	5	6	7	8
*Hippocampus haema* sp. n. (1)	0.000–0.004							
*H. coronatus* (2)	0.025–0.028	0.000–0.001*						
*H. sindonis* (3)	0.075–0.079	0.082	0.000					
*H. mohnikei* (4)	0.104–0.108	0.114–0.115	0.121	–				
*H. kuda* (5)	0.131–0.135	0.139–0.140	0.148	0.110	–			
*H. reidi* (6)	0.134–0.138	0.143–0.144	0.153	0.111	0.020	–		
*H. ingens* (7)	0.131–0.136	0.139–0.140	0.151	0.109	0.031	0.028	–	
*Syngnathus schlegeli* (8)	0.241–0.244	0.247–0.248	0.251	0.232	0.217	0.219	0.231	–
**12S rRNA**	**1**	**2**	**3**	**4**	**5**	**6**	**7**	**8**
*Hippocampus haema* sp. n. (1)	0.000							
*H. coronatus* (2)	0.015	0.000						
*H. sindonis* (3)	0.042–0.046	0.042–0.045	0.000–0.003*					
*H. mohnikei* (4)	0.049–0.052	0.058	0.042–0.052	0.006				
*H. kuda* (5)	0.074	0.074	0.055–0.058	0.039	–			
*H. reidi* (6)	0.074	0.074	0.074–0.078	0.049–0.052	0.055	–		
*H. ingens* (7)	0.068	0.068	0.071–0.074	0.046–0.049	0.055	0.009	–	
*Syngnathus schlegeli* (8)	0.216	0.208	0.204–0.208	0.211–0.213	0.195	0.180	0.191	–

### Key to species of the genus *Hippocampus* in Korea and Japan

**Table:** 

1	No lump on bony body; double gill openings; 10–11 trunk rings	**2**
–	Reddish lumps on fleshy body; single gill opening; 12 trunk rings	***Hippocampus bargibanti* Whitley, 1970**
2	11 trunk rings	**3**
–	10 trunk rings	**6**
3	Blunt spine or no spine on body	**4**
–	Sharp spine on body	***Hippocampus histrix* Kaup, 1856**
4	One blunt cheek spine; trapezoid-shape coronet; no dorsal spot	**5**
–	Two blunt cheek spines; moderately high triangle-shape coronet; no dorsal spot	***Hippocampus mohnikei* Bleeker, 1853**
–	One recurved and sharp cheek spine; very low triangular coronet (degenerative coronet); three dorsal spots (on the 1^st^, 4^th^, and 7^th^ trunk rings) but sometimes absent	***Hippocampus trimaculatus* Leach, 1814**
5	Wide body; 34–38 (36) tail rings	***Hippocampus kuda* Bleeker, 1852**
–	Narrow body; 39–41 (40) tail rings	***Hippocampus kelloggi* Jordan & Snyder, 1901**
6	Four tips on corona flat (5^th^ tip degenerated, and separated from the other four); wing-tip spine on dorsal fin base	**7**
–	Five tips on corona flat (5^th^ tip developed, and combined with the other four); no wing-tip spines on dorsal fin base	***Hippocampus sindonis* Jordan & Snyder, 1901**
7	37–40 (39) tail rings; coronet height from gill opening 43.0–60.1 % head length; coronet height from mid-point of cleithral ring 55.7–79.0 % head length	***Hippocampus coronatus* Temminck & Schlegel, 1850**
–	35–38 (36) tail rings; coronet height from gill opening 22.7–41.6 % head length; coronet height from mid-point of cleithral ring 34.1–54.9 % head length	***Hippocampus haema* sp. n.**
	*This key was compiled from Lourie et al. (1999, 2004), Senou (2013), Lourie (2016), and the current study data.

## Supplementary Material

XML Treatment for Hippocampus coronatus

XML Treatment for Hippocampus sindonis

XML Treatment for Hippocampus haema
