# Corrigenda: Han S-Y, Kim J-K, Kai Y, Senou H (2017) Seahorses of the *Hippocampus coronatus* complex: taxonomic revision, and description of *Hippocampus haema*, a new species from Korea and Japan (Teleostei, Syngnathidae). ZooKeys 712: 113–139. https://doi.org/10.3897/zookeys.712.14955

**DOI:** 10.3897/zookeys.753.25599

**Published:** 2018-04-26

**Authors:** Sang-Yun Han, Jin-Koo Kim, Yoshiaki Kai, Hiroshi Senou

**Affiliations:** 1 Department of Marine Biology, Pukyong National University, Busan, South Korea; 2 Maizuru Fisheries Research Station, Field Science Education and Research Center, Kyoto University, Nagahama, Maizuru, Kyoto, Japan; 3 Kanagawa Prefectural Museum of Natural History, Kanagawa, Japan

Several typing errors need attention and correction:

**Accession numbers in Table 1, page 119:**

In the cyt *b* row of *H. coronatus*, ‘KT167545–KP167548’ should be: KT167545–KT167548.

In the 16S rRNA row of *H. coronatus*, ‘KT167549–KP167552’ should be: KT167549–KT167552.

In the 12S rRNA row of *H. coronatus*, ‘KT167553–KP167556’ should be: KT167553–KT167556.

In the cyt *b* row of *H. sindonis*, ‘KT167539–KP167540’ should be: KT167539–KT167540.

In the 16S rRNA row of *H. sindonis*, ‘KT167541–KP167542’ should be: KT167541–KT167542.

In the 12S rRNA row of *H. sindonis*, ‘KT167543–KP167544’ should be: KT167543–KT167544.

**Table 2, page 121:** The first “Lourie et al. (1999)” column under *H. sindonis* should be moved under *H. coronatus*.

**Material examined of *H. sindonis*, page 127, line 36:** ‘FAKU 137339, 1 93.0 mm’ should be: FAKU 137339, 1, 93.0 mm.

**Description of *H. sindonis*, page 128, line 15:** ‘CoT, 5’ should be: CoT 5.

**Paratypes of *H. haema*, page 130, line 4:** Size range of paratype series should be: 15.9–113.9 mm SL. **Page 130, line 13:** ‘PKU 9713–9717, 9719–9720, 7, 61.6–85.1 mm SL’ should be: PKU 9713–9720, 8, 49.5–85.1 mm SL. **Page 130, line 25:** ‘PKU 11170–11180, 11, 74.2–102.4 mm SL’ should be: PKU 11169–11180, 12, 74.2–102.4 mm SL.

**Discussion, page 134, line 16:** ‘Overfishing could potentially threat *H. haema*’ should be: Overfishing could potentially threaten *H. haema*.

**Acknowledgements, page 135, line 11:** ‘Haecheonma’ should be: Haechunma Co., Ltd.

